# Y_682_G Mutation of Amyloid Precursor Protein Promotes Endo-Lysosomal Dysfunction by Disrupting APP–SorLA Interaction

**DOI:** 10.3389/fncel.2015.00109

**Published:** 2015-04-07

**Authors:** Luca Rosario La Rosa, Lorena Perrone, Morten Schallburg Nielsen, Pietro Calissano, Olav Michael Andersen, Carmela Matrone

**Affiliations:** ^1^Institute of Cellular Biology and Neurobiology, National Council of Research of Rome, Rome, Italy; ^2^Laboratory CiMoTheMA EA 3808, Faculty of Medicine and Pharmacy, University of Poitiers, Poitiers, France; ^3^Department of Biomedicine, Aarhus University, Aarhus, Denmark; ^4^European Brain Research Institute, Rome, Italy; ^5^Danish Research Institute of Translational Neuroscience (DANDRITE), Nordic-EMBL Partnership, Aarhus, Denmark

**Keywords:** APP, Y_682_G mutation, SorLA, Alzheimer’s disease, neuronal trafficking, lysosomes, endosome, Abeta

## Abstract

The intracellular transport and localization of amyloid precursor protein (APP) are critical determinants of APP processing and β-amyloid peptide production, thus crucially important for the pathophysiology of Alzheimer’s disease (AD). Notably, the C-terminal Y_682_ENPTY_687_ domain of APP binds to specific adaptors controlling APP trafficking and sorting in neurons. Mutation on the Y_682_ residue to glycine (Y_682_G) leads to altered APP sorting in hippocampal neurons that favors its accumulation in intracellular compartments and the release of soluble APPα. Such alterations induce premature aging and learning and cognitive deficits in APP Y_682_G mutant mice (APP*^YG/YG^*). Here, we report that Y_682_G mutation affects formation of the APP complex with sortilin-related receptor (SorLA), resulting in endo-lysosomal dysfunctions and neuronal degeneration. Moreover, disruption of the APP/SorLA complex changes the trafficking pathway of SorLA, with its consequent increase in secretion outside neurons. Mutations in the SorLA gene are a prognostic factor in AD, and changes in SorLA levels in cerebrospinal fluid are predictive of AD in humans. These results might open new possibilities in comprehending the role played by SorLA in its interaction with APP and in the progression of neuronal degeneration. In addition, they further underline the crucial role played by Y_682_ residue in controlling APP trafficking in neurons.

## Introduction

Amyloid precursor protein (APP) has been extensively studied for its role as the precursor of β-amyloid peptide (Aβ) in Alzheimer’s disease (AD). However, its physiological function still remains largely enigmatic.

Amyloid precursor protein is a transmembrane protein that is actively sorted among numerous compartments in the cell. After its synthesis, the newly translated APP polypeptide undergoes several post-translational modifications in the Golgi apparatus, including N- and O-glycosylation, sulfation, phosphorylation, and palmitoylation (Guo et al., 2012a; Jiang et al., 2014). Mature APP is then trafficked to the cell surface. Once APP reaches the cell surface, it is rapidly internalized and subsequently trafficked through endocytic and recycling compartments back to the cell surface or degraded in the lysosome (Guo et al., 2012a; Jiang et al., 2014).

Amyloid precursor protein trafficking in neurons is largely controlled by the Y_682_ENPTY_687_ domain via its specific binding to numerous cytosolic adaptor proteins (Russo et al., 2002; Tarr et al., 2002; Zhou et al., 2004, 2009; Tamayev et al., 2009; Muller and Zheng, 2012). In particular (tyrosine-682), Y_682_ modulates the interaction with adaptor proteins through its phosphorylation and dephosphorylation, suggesting that this residue functions as a switch that activates certain APP signaling pathways (Russo et al., 2002; Tarr et al., 2002; Zhou et al., 2004, 2009; Tamayev et al., 2009; Muller and Zheng, 2012). Relevantly, the trafficking of APP affects two competitive enzymatic APP processing pathways, mediated by α- and β-secretases. Notably, an imbalance to the β-secretase-mediated cleavage of APP leads to the accumulation of neurotoxic fragments, including Aβ peptide, which is considered a major cause of AD (Guo et al., 2012a; Jiang et al., 2014).

We previously reported that substituting Y_682_ with a glycine in knock-in animals (APP*^YG/YG^*) promotes a premature, age-dependent decline in cognition, learning, and locomotor performance (Matrone et al., 2012). The Y_682_G mutation induces a redistribution of APP inside neurons and its concomitant accumulation in intracellular compartments (Barbagallo et al., 2010; Matrone et al., 2011; Matrone, 2013). Additionally, the lysosome area is enlarged, and the number of lysosomes is significantly decreased in dorsal root ganglia and fibroblasts from APP*^YG/YG^* mice (Basso and Matrone, 2013; Matrone, 2013).

We believe that Y_682_ plays a crucial role in APP trafficking by possibly controlling APP binding to adaptors and consequently preventing alteration in its cleavage and sorting, finally resulting in neuronal degeneration and death.

Notably, aberrant APP trafficking and deficits in the endo-lysosomal system have been largely described as early causes of neurodegeneration and AD (Nixon et al., 2005; Yu et al., 2005; Lee and Landreth, 2010; Nixon and Yang, 2011). Additionally, aging, a major risk factor for AD, is also associated with defects in autophagocytosis (Cuervo, 2008; Salminen and Kaarniranta, 2009).

Here, we report that Y_682_G mutation induces alternative APP trafficking toward late endosomes (LEs) and lysosomes, ensuing functional alterations of the lysosomal system. Additionally, sortilin-related receptor (SorLA), which controls the trafficking of APP between the trans-Golgi network (TGN) and early endosomes (EEs) by interacting with specific cytosolic adaptors (Willnow and Andersen, 2013), fails to bind mutated APP and is largely secreted outside neurons. Notably, defects in the binding of SorLA and its interacting adaptors are considered important risk factors for AD (Guo et al., 2012b).

These results underline the relevance of residue Y_682_ in controlling APP trafficking and sorting in neurons by likely interacting with SorLA. In addition, they further emphasize the role of SorLA as a potential target protein to monitor APP activity in neurons. Finally, they point to the importance of identification of further adaptors to better understand the molecular events described here.

## Results

### Mutated APP is sorted to the lysosome and late endosome

To analyze the role of Y_682_ in controlling APP trafficking and sorting, we first determined the cellular compartment where mutated APP is preferentially localized using hippocampal neurons from APP*^YG/YG^* and control mice.

We previously reported the accumulation of APP in intracellular compartments and alterations in the number and size of lysosomes from APP*^YG/YG^* neurons (Matrone et al., 2011, 2012). Caster et al. recently reported that APP mutation on Y_682_ preferentially accumulated in lysosomes from HeLa cells (Caster and Kahn, 2013). Therefore, we began our analysis by focusing on the endo-lysosomal compartments of hippocampal neurons from wildtype (WT) and APP*^YG/YG^* mice.

**Figure 1:**
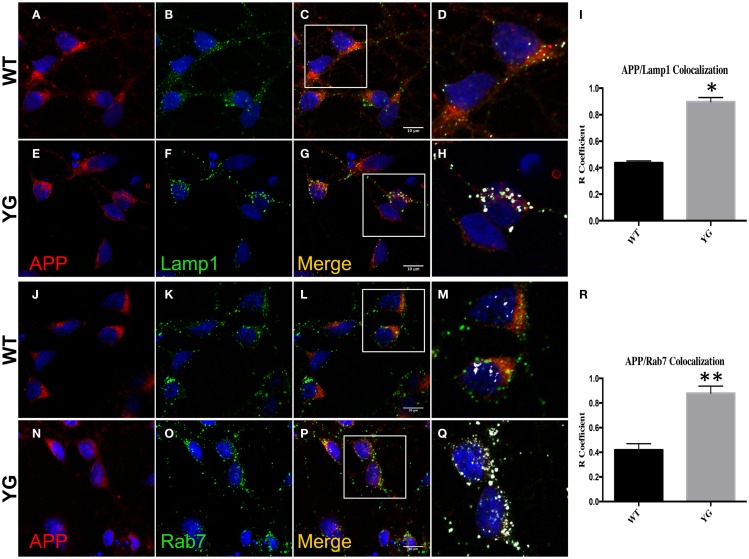
**Amyloid precursor protein increases in LAMP1- and Rab7-positive vesicles**. **(A–G)** Confocal microscopy analysis of double-staining of mouse anti-APP [red; **(A,E)**] and rabbit anti-Lamp1 [green; **(B,F)**] in WT and APP*^YG/YG^* (YG) hippocampal neurons [**(A–D)** and **(E–H)**, respectively; 63× objective). Scale bars = 10 μm. The panels are representative of five different experiments performed in duplicate. [**(C,G)** and **(D,H)**] Co-localization analysis of WT and APP*^YG/YG^* hippocampal neurons. The analysis was performed using Zen/Zeiss software. Notice that the area of overlap between APP and Lamp1 immunostaining (white) was significantly increased in APP^YG/YG^ mice **(H)** compared with WT **(D)**. **(D,H)** High magnification of **(C,G)**. The **(R)** coefficient (Pearson’s coefficient) was used for the quantitative and comparative analyses **(I)**. The data are expressed as mean ± SEM. *n* = 10. *p < 0.05. **(J–P)** Confocal microscopy analysis of double-staining for mouse anti-APP [red; **(J,N)**] and rabbit anti-Rab7 [green; **(K,O)**] in WT and APP*^YG/YG^* (YG) hippocampal neurons [**(J–M)** and **(N–Q)**, respectively; 63× objective]. Scale bars = 10 μm. The panels are representative of four different experiments performed in duplicate. [**(L,P)** and **(M,Q)**] Co-localization analysis of WT and APP*^YG/YG^* hippocampal neurons. The analysis was performed using Zen software. Notice that the area of overlap between APP and Rab7 immunostaining (white) was significantly increased in APP*^YG/YG^* mice **(Q)** compared with WT **(M)**. **(M,Q)** High magnification of **(L,P)**. The **(R)** coefficient (Pearson’s coefficient) was used for the quantitative and comparative analyses **(R)**. The data are expressed as mean ± SEM. *n* = 8. *p < 0.05.

We performed confocal microscopy of neurons that were stained with antibodies against APP and either lysosomal-associated membrane protein 1 (Lamp1) or Rab7 (Figure 1). We found an augmented overlap between the localization of APP (red) and Lamp1 (green) in APP*^YG/YG^* neurons (YG) compared with WT cells (Figure 1I). We also detected an increase in the co-localization between APP and Rab7, a marker of the LE (Figure 1R).

This increase in APP localization inside Lamp1- and Rab7-positive vesicles suggested that the Y_682_G mutation disturbed the recycling of APP to the plasma membrane (PM), triggering its accumulation in endo-lysosomal compartments.

**Figure 2:**
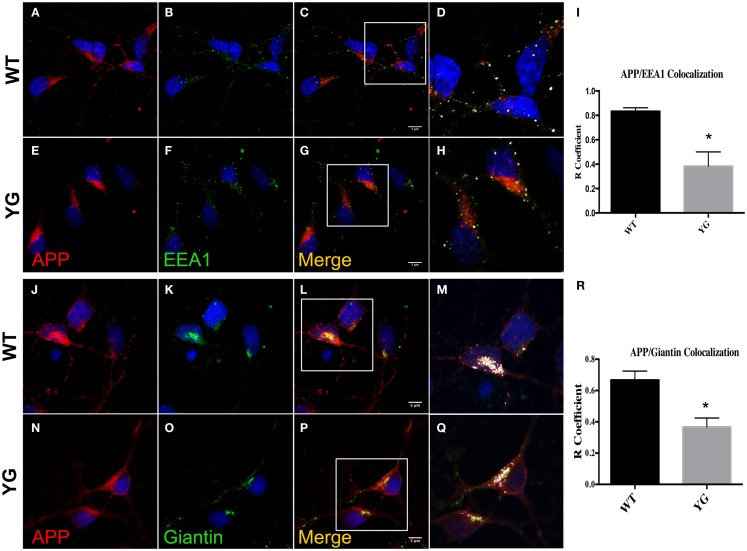
**Amyloid precursor protein decreases in Golgi compartment and early endosomes**. **(A–G)** Confocal microscopy analysis of double-staining for rabbit anti-APP red; **(A,E)**] and mouse anti-EEA1 [green; **(B,F)**] in WT and APP*^YG/YG^* (YG) hippocampal neurons [**(A–D)** and **(E–H)**, respectively; 63× objective]. Scale bars = 7 μm. The panels are representative of three different experiments performed in duplicate. **(C,G)** and **(D,H)** Co-localization analysis of WT and APP*^YG/YG^* hippocampal neurons. The analysis was performed using Zen software. Notice that the area of overlap between APP and EEA1 immunostaining (white) was significantly decreased in APP^YG/YG^ mice **(H)** compared with WT **(D)**. The *R* coefficient (Pearson’s coefficient) was used for the quantitative and comparative analyses **(I)**. The data are expressed as mean ± SEM. *n* = 6. **p < 0.001. **(J,P)** Confocal microscopy analysis of double-staining for rabbit anti-APP [red; **(J,N)**] and mouse anti-giantin [green; **(K,O)**] in WT and APP^YG/YG^ (YG) hippocampal neurons [**(J–L)** and **(N–P)**, respectively; 63× objective]. Scale bars = 5 μm. The panels are representative of five different experiments performed in duplicate. **(L,P)** and **(M,Q)** Co-localization analysis of WT and APP*^YG/YG^* hippocampal neurons. The analysis was performed using Zen software. The **(R)** coefficient (Pearson’s coefficient) was used for the quantitative and comparative analyses **(R)**. The data are expressed as mean ± SEM. *n* = 10. **p < 0.01.

We next examined whether APP trafficking to the EE and Golgi compartment was also altered in APP*^YG/YG^* neurons. Interestingly, immunostaining for EEA1, a marker of EEs, indicated a reduction of co-localization with mutated APP in hippocampal neurons (Figures 2A–I). Similarly, APP localization in Golgi compartments that were positive for (Figures 2M,Q,R) Giantin was also reduced by the Y_682_G mutation (Figures 2J–R).

### Y_682_ mutation affects lysosomal activity in hippocampal YG neurons

**Figure 3:**
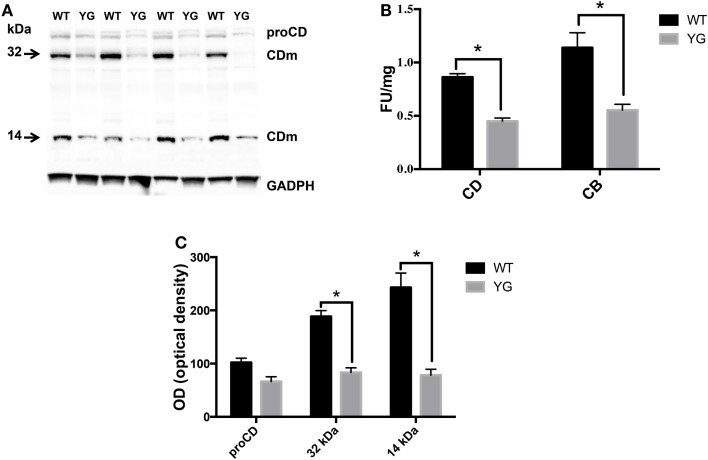
**Lysosome activity is impaired in APP^YG/YG^ hippocampal neurons**. **(A)** Western blot analysis of cathepsin D in hippocampi from WT and APP*^YG/YG^* (YG) mice and **(B)** the corresponding densitometric analysis. The data were normalized to the corresponding GADPH values. The optical density (OD) is expressed as a percentage (mean ± SEM) of the proCD values from WT samples. *n* = 3. *p < 0.05. **(C)** Cathepsin D (CD) and cathepsin B (CB) cysteine protease activity in WT and APP^YG/YG^ (YG) hippocampal neurons. The samples were collected and analyzed according to the procedure suggested by the manufacturer and described in the Section “Materials and Methods.” The relative activity is expressed as fluorescent units (FU)/mg protein. The data are expressed as mean ± SEM. *n* = 3. *p < 0.05.

We next investigated whether the observed accumulation of APP in Lamp1-positive vesicles leads to a defect in lysosomal activity. To test for lysosomal activity, we used western blotting (WB) to detect the conversion of immature to mature cathepsin D (CD), a lysosomal protease that is used as an indirect marker of lysosomal function (Luzio et al., 2007). The WB analysis of homogenates from WT and APP*^YG/YG^* neurons showed a significant decrease in the level of the two CD active fragments, which migrate at 32 and 14 kDa (CDm; Figures 3A,B). Furthermore, the enzymatic activity of CD and cathepsin B (CB) confirmed a significant reduction of lysosomal activity in neurons isolated from APP*^YG/YG^* mice (Figure 3C). These data clearly suggest impairment in the lysosomal machinery and a key role played by the Y_682_ENPTY_687_ domain in controlling APP trafficking in neurons and preventing lysosomal dysfunction. These results are also interesting when considering that a reduction of CD activity has been previously reported in brains from AD patients (Yang et al., 2011).

### Y_682_G mutation affects APP binding to SorLA and increases SorLA secretion outside neurons

The Y_682_ENPTY_687_ motif within the APP cytoplasmic domain plays a crucial role in the sorting and trafficking of APP through its binding to specific adaptor proteins. The recruitment of specific adaptors to APP is linked to its routing throughout the endomembrane system and its phosphorylation state, particularly the Y_682_ residue (Russo et al., 2002; Tarr et al., 2002; Zhou et al., 2004, 2009; Tamayev et al., 2009; Muller and Zheng, 2012).

The Vps10p-D receptor, SorLA, is a neuronal protein that shuttles from the PM to endosomes and TGN and regulates the trafficking and processing of APP (Andersen et al., 2005). SorLA confines APP to the Golgi apparatus and affects its transition to the cell surface, a step that is crucial for conversion to both soluble APPα (sAPPα) and sAPPβ/Aβ (Andersen et al., 2005, 2006; Dodson et al., 2008; Fjorback et al., 2012). We observed the enhancement of mutated APP trafficking to Rab7-positive vesicles and sought to determine whether Y_682_G substitution impairs the binding of APP to SorLA.

**Figure 4:**
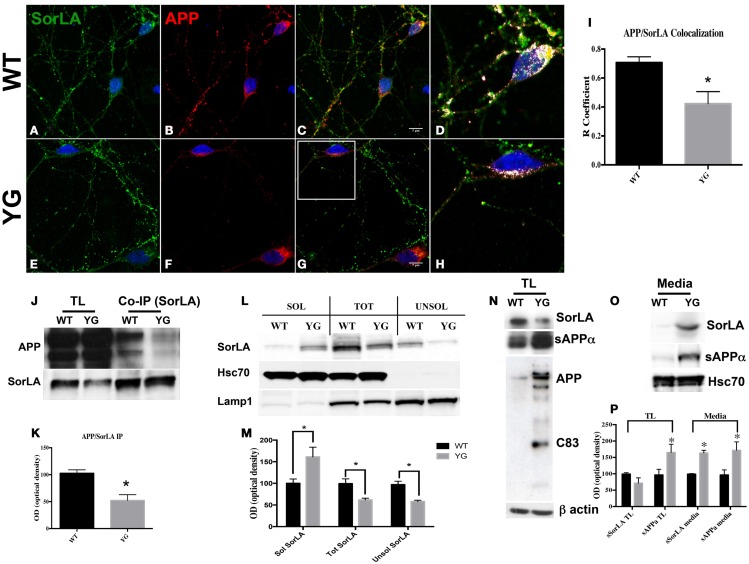
**Y_682_G mutation prevents binding of APP to SorLA and increases SorLA secretion**. **(A–H)** Confocal microscopy analysis of double-staining for rabbit anti-SorLA [green; **(A,E)**] and mouse anti-APP [red; **(B,F)**] in WT and APP*^YG/YG^* (YG) hippocampal neurons [**(A–C)** and **(E–G)**, respectively; 63× objective]. Scale bars = 7 μm. **(D,H)** high magnification of the **(C)** and **(G)**, respectively. Co-localization analysis was performed using Zen software **(I)**. The panels are representative of seven different experiments. **(G)** Quantitative analysis was performed by comparing the *R* coefficient (Pearson’s coefficient) between WT and APP*^YG/YG^* (YG) neurons. *n* = 7. *p < 0.05. **(J,K)**. Protein samples from hippocampi from either WT or APP*^YG/YG^* (YG) mice were immunoprecipitated with mouse anti-α-SorLA antibody (co-IP SorLA) and analyzed with rabbit anti-APP. TL, total lysate; IP, immunoprecipitate. The same membrane was stripped and analyzed with mouse anti-SorLA. **(L,M)** SorLA WB analysis in the soluble and insoluble fractions from hippocampal tissues from WT and APP*^YG/YG^* (YG) mice. As positive and negative controls for the fractionations, we used HSC70 protein and Lamp1, respectively. The optical density analysis is reported below. Data from the soluble (Sol) and insoluble (Unsol) fractions were normalized to the corresponding HSC70 and Lamp1 values and are expressed as a percentage of WT (*n* = 4). *p < 0.05 vs. WT (Newman–Keuls test). **(N)** WB analysis of APP, sAPPα, and sSorLA from hippocampal primary neuronal cultures of WT and APP*^YG/YG^* (YG) mice **(O)** Medium collected from APP*^YG/YG^* (YG) neurons and the corresponding control (WT) samples were analyzed by WB for sAPPα and sSorLA (see Materials and Methods). WB is representative of three different experiments. **(P)** Densitometric analysis of **(N,O)**. Black histogram: WT; Gray histogram: YG. (*N* = 3; Newman–Keuls test) *p < 0.05 vs. WT.

Confocal microscopy of hippocampal neurons that were immunostained for APP and SorLA showed a strong co-localization between SorLA and APP in WT cells (Figures 4A–D). However, such co-localization was significantly reduced in APP*^YG/YG^* neurons (Figures 4E–H). Interestingly, confocal microscopy analysis indicated a decrease in SorLA in the cell body and an increase along the neurites of Y_682_G neurons (Figure 4).

Consistent with the reduced co-localization, co-immuno precipitation (co-IP) experiments from the hippocampi of WT and APP*^YG/YG^* mice showed a reduced complex formation between APP and SorLA in APP*^YG/YG^* neurons (Figures 4J,K).

The way in which Y_682_ mutation disturbs the binding of SorLA to APP is still unknown. One plausible mechanism is that some adaptor proteins may fail to bind APP or SorLA, resulting in alterations of the APP/SorLA complex.

Sortilin-related receptor has been largely described as a potential tool that is predictive of the progression of AD in humans. In fact, the levels of SorLA appeared to be reduced in AD brain tissues and increased in cerebrospinal fluid (Ikeuchi et al., 2010; Guo et al., 2012b). Therefore, we hypothesized that the lack of formation of the APP/SorLA complex might result in the missorting of SorLA and its secretion outside neurons. To test this hypothesis, we analyzed the protein levels of SorLA in both soluble and insoluble hippocampal fractions from APP*^YG/YG^* mice and relative controls (Figures 4L,M). As expected, SorLA levels in the soluble fraction from APP*^YG/YG^* tissue was significantly increased, whereas its levels appeared to be reduced in the corresponding insoluble fraction (Figures 4L,M). The increased secretion of SorLA from APP*^YG/YG^* neurons was further analyzed in the medium of primary hippocampal neurons. Also here, the amount of SorLA released from APP*^YG/YG^* neurons into the medium was greatly increased when compared to the control samples (Figures 4O,P). Such increase was paralleled by an intensification of sAPPα amount in the medium of APP*^YG/YG^* neurons. Consistently, the corresponding SorLA intracellular levels appeared significantly reduced (Figure 4N). Notably and consistent with the evidence that Y_682_G mutation induces a preferential α-secretase-mediated APP cleavage (Basso and Matrone, 2013), WB analysis performed with anti-sAPPα antibody showed an increase in sAPPα in APP*^YG/YG^* neurons (Figure 4N).

## Discussion

Altogether, our data further elucidate the role played by the Y_682_ENPTY_687_ domain in controlling APP trafficking and sorting in neurons. Our data support a model whereby alterations in the binding of APP to SorLA likely leads to an aberrant misrouting of APP in hippocampal neurons, resulting in APP accumulation in endo-lysosomal compartments and autophagic defects (see Box 1).

Box 1 **Y_682_G mutation alters APP trafficking and leads to an increase in SorLA secretion.**

**Figure:**
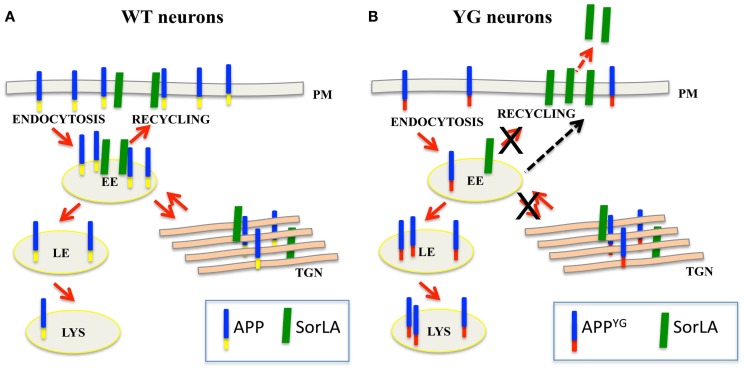
**(A)** In normal neurons, SorLA (green) controls APP (blue–yellow) trafficking from early endosome (EE) back to the trans-Golgi network (TGN) or plasma membrane (PM). **(B)** Because of the Y_682_G mutation on the Y_682_ENPTY_687_ domain of APP (YG), SorLA is no longer able to interact with mutated APP (blue–red); consequently, mutated APP mislocalizes to the late endosome (LE) and lysosome (LS) where it is likely processed to generate CTF peptides (Basso and Matrone, 2013). The accumulation of APP in the late endosome and lysosomes induces alterations in lysosomal activity and leads to the neuronal defects that were previously reported in APP*^YG/YG^* mice (YG) (Matrone, 2013). As a result of the lack of binding to APP, SorLA is trafficked to the plasma membrane where it is rapidly cleaved and secreted outside neurons.

The intracellular portion of APP, which encompasses the Y_682_ENPTY_687_ sequence of AICD, has been reported to interact with several different adaptors. Y_682_ENPTY_687_ plays a crucial role in modulating the binding and unbinding of APP to specific adaptor proteins via phosphorylation and dephosphorylation of the Y_682_ residues. When Y_682_ is phosphorylated, it creates docking sites for cytosolic proteins, such as Shc, Grb2, and Grb7, and inhibits the binding of others, such as the Fe65 family. Differently, when Y_682_ is dephosphorylated, the binding of proteins containing a phosphotyrosine-binding (PTB) domain is impaired (Russo et al., 2002; Tarr et al., 2002; Zhou et al., 2004, 2009; Tamayev et al., 2009; Muller and Zheng, 2012).

Here, we report that when Y_682_ residue is mutated (Y_682_G mutation), APP is misrouted to the LEs/lysosomes, triggering lysosomal defects and contributing to progressive and premature aging and the neurodegenerative phenotype previously reported in YG knock-in mice (Matrone et al., 2012).

In addition, we provide clear evidence of the role played by Y_682_ in the interaction of APP with SorLA, and we speculate on the possibility that the lack of a proper interaction between SorLA and APP could lead to the misrouting of APP in neurons and consequently to the lysosomal defects previously observed in APP*^YG/YG^* mice (Matrone et al., 2012). Interestingly, the lack of APP and SorLA interaction also results in increased secretion of SorLA into the soluble fraction of APP*^YG/YG^* brains and into the medium of cultured hippocampal APP*^YG/YG^* neurons.

The role of SorLA in controlling APP trafficking has been previously clarified (Andersen et al., 2005). SorLA directly binds APP and controls APP trafficking from EEs back to the TGN or PM, thus preventing β- and α-secretase-mediated APP cleavage.

The complexity in the APP and SorLA interaction has been previously predicted in the SorLA-null mouse model and in several SorLA-overexpressing cell lines (Andersen et al., 2005; Spoelgen et al., 2006; Schmidt et al., 2012; Mehmedbasic et al., 2015). All these studies have allowed the identification of numerous putative SorLA binding sites on both the N- and C-terminal sequences of APP depending on the cellular context, pointing to the possibility that APP interacts with SorLA at multiple sites and that such interaction probably requires the additional collaboration of other adaptors (Andersen et al., 2005; Spoelgen et al., 2006; Schmidt et al., 2012; Mehmedbasic et al., 2015).

Accordingly, we report here that the alteration in APP binding to SorLA (and likely to other adaptors) leads to APP accumulation in LEs and lysosomes and to autophagic defects, which are considered a major risk factor for AD and dementia during aging (Cuervo, 2008; Salminen and Kaarniranta, 2009).

In addition, evidence from pathophysiological and genetic studies clearly suggests that SorLA plays a crucial role in AD-related processes. Several studies of brain or cerebrospinal fluid specimens have reported alterations in the levels of SorLA in the brains of some individuals with sporadic AD (Ma et al., 2009; Ikeuchi et al., 2010). Moreover, several mutations of the SorLA gene have been associated with AD (Rogaeva et al., 2007; Alexopoulos et al., 2011; Caglayan et al., 2014).

Based on the critical phenotype previously observed in APP*^YG/YG^* mice, which resulted in premature aging and cognitive and learning deficits (Matrone et al., 2011, 2012), and the potential role of SorLA as a predictive factor of neurodegeneration in AD patients (Rogaeva et al., 2007; Alexopoulos et al., 2011; Caglayan et al., 2014), we believe that these results strongly underscore the relevance of the correct APP binding to adaptor proteins, via Y_682_ENPTY_687_ domain, as a future potential strategy to prevent neurodegeneration *in vivo*.

## Materials and Methods

### Animal model

Male homozygous APP Y_682_G mice (APP*^YG/YG^*) and WT controls (C57BL/6J, approximately 30–40 days of age) were used in this study. The APP*^YG/YG^* mice were a generous gift from Prof. D’Adamio and have been previously described in detail (Barbagallo et al., 2010, 2011; Matrone et al., 2011, 2012; Basso and Matrone, 2013; Matrone, 2013). The mice from both lines were bred and group-housed in the Lab Animal Centre of Taconic (Ejby, DK) at an ambient temperature of 22–23°C and on a 12/12 h dark/light cycle (lights on 7 a.m.).

All of the mice were anesthetized by 2-bromo-2-chloro-1,1,1-trifluoroethane inhalation and euthanized in accordance with international guidelines on the ethical use of animals (European Communities Council Directive of November 24, 1986; 86/609/EEC) and Danish guidelines. The brains were quickly removed from the skulls, and the hippocampi were dissected on ice according to the procedure previously described by La Rosa et al. (2013).

We previously reported that neuronal decline in APP*^YG/YG^* mice starts to be evident at 3 months (Matrone et al., 2012), for this reason all of the experiments reported herein were performed in 3-month-old APP^YG/YG^ and control mice.

### Immunoprecipitation

For the IP reactions, protein samples were added to Dynabeads-Protein G (30 μg/100 μl) according to the procedure described by the manufacturer (Invitrogen, Naerum, Denmark) and eluted with 0.1 M citrate buffer (pH 2.3).

For the IP analysis, we used anti-mouse anti-SorLA from Abcam [mouse monoclonal (3B6B11) to SorLA]. The WB analysis was performed using rabbit anti-APP antibody (clone Y188) from Abcam.

### Western blot analysis

Whole mouse brain was sonicated in a buffer containing 20 mM Tris-base pH 7.4, 250 mM sucrose, 1 mM EDTA, 1 mM EGTA plus protease (Roche, Complete) and phosphatase inhibitors.

To obtain soluble and insoluble fractions, the lysates were first spun at 1,000 × *g* for 15 min and then for an additional 4 h at 100,000 × *g*. The soluble fraction was separated from the pellet and analyzed by WB. Equal amounts (30 μg) of proteins were separated on 4–12% Bis-Tris SDS-PAGE gels (Invitrogen, DK), blotted onto nitrocellulose membranes (Amersham, DK), and incubated overnight with the appropriate primary antibody. We used rabbit anti-cathepsin D and rabbit anti-Lamp1 from Santa Cruz (Santa Cruz, CA, USA, DK); mouse anti-SorLA (clone 3B6B11) and rabbit anti-APP (clone Y188) from Abcam (Abcam, UK).

Media collected from APP*^YG/YG^* neurons and the correspondent control samples were centrifuged at 10,000 × *g* for 30 min to remove cell debris, and the supernatant was further centrifuged at 100,000 × *g* for 12 h at 4°C. The pellets were solubilized in 2% SDS-loading buffer and analyzed by WB for sAPPα and sSorLA. To detect α-secretase-mediated APP cleaved peptides, we used anti α-APP (MyBioSource, clone MBS533522). An antibody against the N-terminal domain of SorLA (sc33822) from Santa Cruz Biotechnology (Santa Cruz, CA, USA) was used to assess the amount of SorLA released into the medium and retained in neurons.

### Primary hippocampal neuronal cultures

Hippocampal neurons were prepared from embryonic day 17/18 (E17/18) mice as previously reported (Matrone et al., 2008, 2009). The hippocampi were dissected in Hank’s Balanced Salt Solution buffered with HEPES and dissociated via trypsin/ethylenediaminetetraacetic acid treatment. A total of 10^6^ cells were plated on 3.5-cm dishes that were precoated with poly-l-lysine. After 2 days of culture in neurobasal medium that contained B-27 supplement and Glutamax, cytosine arabinofuranoside was added to reduce glial proliferation.

### Confocal microscopy and co-localization analysis

The neurons were fixed for 20 min in phosphate-buffered saline (PBS) that contained 4% formaldehyde, permeabilized with 0.05% Triton (5–10 min for 20°C), and processed for double-labeling with the appropriate antibodies. Secondary antibodies coupled to Alexa dyes (488 or 594) were obtained from Abcam (Cambridge, UK). The nuclei were visualized by staining with DAPI (1 μg/ml; Sigma Brøndby, Denmark). Digital images were obtained with a Zeiss LSM confocal lsm780 system using 63× oil NA 1.3 objective. Quantification of the co-localization experiments was performed using Zen 2009 software. Pearson coefficients (*R* coefficients) were used as co-localization coefficients.

For the immunofluorescence analysis, we used rabbit anti-Lamp1 (sc199992), rabbit anti-APP (clone Y188), mouse anti-Rab7 (Rab7-117), mouse anti-EEA1 [anti-EEA1 antibody (1G11); EE marker], and mouse anti-giantin (clone 9B6), from Abcam. For the experiments reported in Figures 4A–H,N,O we used anti α-APP (MyBioSource, clone MBS533522) and anti-SorLA (sc33822) from Santa Cruz Biotechnology (Santa Cruz, CA, USA).

### Measurement of cathepsin D and B activity

Enzymatic CD and CB activity was examined in hippocampal tissues from WT and YG mice using a CD or CB activity assay kit (Abcam). Briefly, tissues were washed twice in ice-cold PBS and then homogenized in extraction buffer. After 10 min incubation on ice, the extract was centrifuged at 10,000 × *g* for 5 min, and 50 μl of the supernatant was mixed with an equal volume of 2× reaction buffer and 2 μl substrate (GKPIFFRLK[Dnp]-DR-NH_2_-MCA substrate for CD or 10 mM Ac-RR-AFC substrate for CB) in a 96-well microplate. The plates were kept in the dark at 37°C for 1 h, and fluorescence was recorded using a FLUOstar Optima plate reader (BMG Labtech, Milan, Italy). The protein concentration was determined by the BCA method (Bio-Rad Laboratories, Hercules, CA, USA) at 328 and 460 nm excitation and emission wavelengths, respectively, for CD activity or 400 and 505 nm excitation and emission wavelengths for CB activity.

### Statistical analysis

The statistical analysis was performed using two-tailed *t*-tests, and multiple comparisons were made using one-way, two-way, or two-way repeated-measures analysis of variance (ANOVA) followed by a *post hoc* test using GraphPad Prism 6 software (San Diego, CA, USA). Statistical significance was accepted at the 95% confidence level (*p* < 0.05).

## Author Contributions

LLR and CM designed the study and performed the experiments; MN, OA, and CM interpreted and analyzed the data; MN, LP, and PC provided critical discussion about biological significance; OA and CM wrote the paper.

## Conflict of Interest Statement

The authors declare that the research was conducted in the absence of any commercial or financial relationships that could be construed as a potential conflict of interest.

## Abbreviations

Aβ, amyloid beta; APP, amyloid precursor protein; CTF, carboxyl terminal fragment; EEA1, early endosome-associated protein 1; Lamp1, lysosomal-associated membrane protein 1; sAPPα, soluble amyloid precursor protein alpha; sAPPβ, soluble amyloid precursor protein beta; IF, immunofluorescence; IP, immunoprecipitation; SorLA, sortilin-related receptor; TL, total lysate; WB, western blot; WT, wildtype; YG, mutated amyloid precursor protein: APP Y_682_G.
